# Dr. A. R. Wallace and the Vaccination Commission

**Published:** 1891-09-05

**Authors:** 


### EDITOR'S LETTER-BOX.

[Our correspondents are reminded that prolixity is a great bar to publication, and that brevity of style and conciseness of statement greatly facilitate early insertion].

### DR. A. R. WALLACE AND THE VACCINATION COMMISSION.

Sir,—Your journal has hitherto been so honourably distinguished among the medical press for its fairness to the anti-vaccinists that I the more regret the recent appearance in its columns of an editorial paragraph adversely criticising Dr. Wallace. I have read the official reports myself of the Commission, but am inclined to think that your contributor has taken his data at second-hand. Dr. Wallace himself anticipated your criticism, and, in one of the noblest statements ever made by a scientific man in defence of right and justice, completely deprives it of any weight. But even discounting his evidence to the fullest extent, and by the most hostile interpretation, there yet remains sufficient in it to absolutely destroy all justification for imposing this medical dogma by law upon the nation. As to taking up a position of what you term "pure science" on the matter, the notion is little short of absurd, as from first to last the whole thing is a mass of contradictions, no two "authorities"—save the mark!—scarcely agreeing on one single point. Even to this hour vaccination is not either scientifically or legally defined. Jenner himself, as is well known, died an equinator, thus finally throwing over the cow altogether. And it is a common-place that since his day until about 1840 the thing was taken on trust, and even after Ceely's and Badcock's experiments at that time until Creighton's and Crookshank's recent independent investigations, it was still taken on trust. If the medical profession had a proper regard for their permanent interests, they would hasten to dissociate themselves from vaccination. Indeed, the time seems to be approaching when the expression of a belief in vaccination by a medical man will be deemed a bar to his employment. Opinion is clearly moving in this direction. Professor Creighton, like his medical brethren, believed in vaccination until it became his duty, as a pathologist at Cambridge University, to investigate it. Then he found it to be a delusion! This is how he speaks of the "pure science" in vaccinal matters of medical orthodoxy. "But what shall we say of pathology, which has not the candour even to recognise the juxtaposition of incompatible notions, which can show no better front to the world than a thin tissue of rhetoric or metaphor made to do duty as scientific authority, which shelters itself, wherever it can, behind the establishment by law of its own doctrine deliberately left undefined and unformulated?" And Crookshank, as it is said, set on to check Creighton, came to practically an identical conclusion. Jenner, he said, misled the medical profession. I would not waste a moment in writing to the so-called "great" medical papers. It would be as useless as to write to orthodox Church papers criticising the doctrine of the Trinity. They are more the organs of professional vested interests than honest public instructors. But in The Hospital I have noticed a different tone—a higher morality. It is alive, progessive, modern, and I cannot think it will for long support the rapidly discredited doctrine of vaccination. If it does, so much the worse for it, as the signs are clear to those who will see that the people are taking this thing into their own hands, and that compulsion, at any rate, is doomed despite the grossly packed Royal Commission.—Yours obediently, J. H.

August 14, 1891.

[This letter is dealt with at page 272.—Ed.]

